# Risk factors for maternal death in the highlands of rural northern Tanzania: a case-control study

**DOI:** 10.1186/1471-2458-8-52

**Published:** 2008-02-08

**Authors:** Bjørg Evjen-Olsen, Sven Gudmund Hinderaker, Rolv Terje Lie, Per Bergsjø, Peter Gasheka, Gunnar Kvåle

**Affiliations:** 1 Haydom Lutheran Hospital, Mbulu District, Manyara Region, Tanzania; 2 Centre for International Health, Armauer Hansen Building, University of Bergen, NO-5021 Bergen, Norway; 3 Department of Public Health and Primary Health Care, Section for Epidemiology and Medical Statistics, University of Bergen, NO-5021 Bergen, Norway; 4 Division of Epidemiology, Norwegian Institute of Public Health, PO Box 4404 Nydalen, NO-0403 Oslo, Norway

## Abstract

**Background:**

Tanzania has one of the highest maternal mortality ratios in sub-Saharan Africa. Due to the paucity of epidemiological information on maternal deaths, and the high maternal mortality estimates found earlier in the study area, our objective was to assess determinants of maternal deaths in a rural setting in the highlands of northern Tanzania by comparing the women dying of maternal causes with women from the same population who had attended antenatal clinics in the same time period.

**Methods:**

A case-control study was done in two administrative divisions in Mbulu and Hanang districts in rural Tanzania. Forty-five cases of maternal death were found through a comprehensive community- and health-facility based study in 1995 and 1996, while 135 antenatal attendees from four antenatal clinics in the same population, geographical area, and time-span of 1995–96 served as controls. The cases and controls were compared using multivariate logistic regression analyses. Odds ratios, with 95% confidence intervals, were used as an approximation of relative risk, and were adjusted for place of residence (ward) and age. Further adjustment was done for potentially confounding variables.

**Results:**

An increased risk of maternal deaths was found for women from 35–49 years versus 15–24 years (OR 4.0; 95%CI 1.5–10.6). Women from ethnic groups other than the two indigenous groups of the area had an increased risk of maternal death (OR 13.6; 95%CI 2.5–75.0). There was an increased risk when women or husbands adhered to traditional beliefs, (OR 2.1; 95%CI 1.0–4.5) and (OR 2.6; 95%CI 1.2–5.7), respectively. Women whose husbands did not have any formal education appeared to have an increased risk (OR 2.2; 95%CI 1.0–5.0).

**Conclusion:**

Increasing maternal age, ethnic and religious affiliation, and low formal education of the husbands were associated with increased risk of maternal death. Increased attention needs to be given to formal education of both men and women. In addition, education of the male decision-makers should be given high priority in the community, especially in matters concerning pregnancy and delivery preparedness, since their choice greatly affects the survival of the pregnant and delivering women.

## Background

Maternal mortality has recently been included among the Millennium Development Goals (MDG 5). In spite of national efforts, Tanzania has one of the highest maternal mortality ratios (MMR) in Sub-Saharan Africa, with national estimates as high as 1100 in 1995 at the time of the present study, increasing to 1500 per 100 000 live births in 2006 [1,2].

Loudon noted how maternal deaths throughout history have been, and still are, devastating in a way that other causes of death are not [3]. He emphasised how maternal care and maternal deaths cannot be divided into neat compartments categorised as social, political, economic, demographic, professional or clinical. His historical and statistical overview reveals the complexity and multi-factorial aspects of maternal mortality. Individual risk factors such as age, parity, and education, among others, may only be markers for groups of women at increased risk, rather than direct causes of poor outcome, and hence, poor predictors of risk [4]. Presently, it is acknowledged that the ability to discriminate between women at high and low risk in all formal risk-scoring systems is poor. Consequently, all pregnant women are at risk of maternal death. Thus, some of the main international strategies to reduce maternal mortality are operational rather than risk oriented, such as improving the quality and availability of emergency obstetric facilities and increasing the number of births attended to by a skilled attendant [5,6]. In order to influence and develop public health strategies in the quest for reducing the high mortality rates in many developing countries, research on risk factors is still valid.

Several studies have been done on maternal mortality and on the assessment of risk factors for maternal deaths in Tanzania [7-14] and elsewhere [15-20]. These have revealed that age, parity, education of mothers, obstetric factors, unavailability of health facilities and trained health personnel, and socio-economic factors, are associated with an increased risk of maternal death.

There is a beginning transition to the more "modern" society in some parts of the study area, bringing societal and cultural change [21]. On the other hand, people in other parts of this area still adhere to a way of living that has been largely unchanged through generations, with the families being headed by male decision-makers, who have great influence on all matters related to pregnancy and childbirth. Previous research in the study area showed an MMR of 382 per 100 000 live births (95%CI:250–560) [22]. Hence, due to the paucity of epidemiological information on maternal deaths, the cultural and societal conditions, and the high estimated maternal mortality ratio, our objective was to assess determinants of maternal mortality in this rural setting in the highlands of northern Tanzania by comparing the women dying of maternal causes with women who had attended antenatal clinics and had not died.

## Methods

### Study area and target population

The study area was situated in Mbulu and Hanang districts in Arusha region, northern Tanzania. The two administrative divisions of Dongobesh (Mbulu) and Basotu (Hanang) were selected. They constituted the main catchment area of the Haydom Lutheran Hospital (HLH) at the time of the study. These two divisions had a total of 12 wards comprising 42 villages, with distances from the Haydom hospital of up to 100 kilometres. The hospital had an extensive network of antenatal care clinics (ANC), one at the hospital and 11 outreach clinics. The hospital was the only institution offering comprehensive obstetric care (including caesarean section) in the study area.

Based on the 1988 national census, and an annual growth rate of 2.8% nationally and 3.8 % for Arusha region, for 1995 we extrapolated the population for Tanzania to be 28,116,360 [23], for Arusha region 1,754,906 and for the study area (Dongobesh and Basotu divisions) to be 142554 [24]. Further, the population of women of reproductive age in Arusha Region for 1995 was extrapolated to be 385,022 and for the study area 27,737. Finally, with a crude birth rate of 46 nationally and 47.7 found in a household study in this study area [25], the estimated number of live births for Tanzania was 1,293,353, for Arusha Region 80,726 and for the study area 6800 in 1995. The antenatal attendees (2659 women) among whom the control group was selected, constituted approximately 39 % of the expected number of live births in the study area during this period.

### Subjects and methods

We assessed risk factors for maternal death by a case-control approach, comparing cases from a study on maternal deaths in the catchment area of HLH with controls from an antenatal clinic study in the same geographical area, ongoing at the same time. A total of 45 maternal deaths were identified. The cases were identified in a comprehensive health-facility and population-based study on maternal mortality from all the villages and health facilities in Basotu and Dongobesh divisions in 1995 and 1996. Information on the women dying of maternal causes was obtained through verbal autopsies [26] and health facility records, with a medical doctor and a field assistant interviewing a relative of the deceased. Information on demographic and pregnancy-related variables, in addition to a detailed obstetric history, was recorded. Maternal deaths were defined as deaths related to pregnancy and delivery up to 42 days after termination of pregnancy [1]. Sixteen of the respondents were husbands, 17 were from the husband's family, seven were from the woman's own family, and one was a second wife. The verbal autopsies were conducted in the period from 1995 until 1999. The recall period for the cases ranged from approximately three months to four years. The reason for the long recall period in some cases was due to the difficulty in tracing families. This area is remote, and we returned several times in order to find as many as possible. The causes of death among the cases are described in an earlier publication. The diagnoses were set by a panel of four doctors, using all available information. Of 45 total maternal deaths, 13 were direct obstetric deaths, while 32 were indirect. Haemorrhage was the main cause of direct obstetric deaths (five cases). Cerebral malaria accounted for 44.4% of the total number of maternal deaths (20 of 45), all of them labelled as indirect obstetric deaths. Among the malaria cases, 19 were verified by blood slide, while one was diagnosed as malaria being the most probable cause of death, due to clinical information, but not verified by blood slide [27].

The control group was selected in the following manner. In parallel with the study on maternal deaths, in the period February 1995 till March 1996 we also conducted a study on anaemia among antenatal attendees at four of the antenatal clinics run by Haydom Lutheran Hospital. Participants in this study were from the same geographical area and were recruited among the same antenatal population as the study on maternal deaths. In the anaemia study, 2659 women were screened on haemoglobin levels, and 135 of these were selected as controls in the present study. Since the original study was a screening on haemoglobin, and in order to attain the same haemoglobin distribution as in the original population of 2659 women, we used a method of proportional allocation to different categories of haemoglobin levels, calculated in such a way as to ensure that the 135 women could be regarded as a representative, proportional sample of all 2659 women.

None of the women in the control group died within one year after delivery. The women in the control group were interviewed and answered a questionnaire containing similar socio-demographic and obstetric history questions as the relatives of the women who died from maternal causes. Among those who had had two different pregnancies within the time of the study, only information from the first was included, in order to avoid bias. Field assistants in the antenatal clinics interviewed the women in the control group, collecting similar data as among the cases.

### Ethical considerations

The National Committee for Research Ethics in Medicine in Norway and the Commission for Science and Technology in Tanzania (COSTECH) approved the study. The Regional Development Director, the District Commissioners, local ward, division, village and sub-village leaders gave consent to the study, and the local people were informed through village meetings. Each respondent gave individual oral consent.

### Statistical analyses

Data-entry was done using the Epi-Info version 5.0 and 6.0 programs. The Statistical Package for Social Sciences (SPSS) version 9.0 was used for analyses. Odds ratios (OR) were used as an approximation of relative risk, and were given with 95% confidence intervals. Odds ratios for maternal death presented in table 1 were obtained for potential risk factors (age, ethnic and religious affiliation, education, parity, previous abortions, stillbirths, and perinatal deaths), with multivariate logistic regression analyses and adjustment for age and place of residence (ward). Wards were grouped according to geographic location: 1 = Haydom (reference), 2 = Dongobesh+Tumati+Bashay; 3 = Basotu+Basodesh; 4 = Maghang+Maretadu; 5 = Laghanga+Getanwas+Hirbadaw; 6 = Yaeda Chini.

**Table 1:** Risk of maternal deaths by demographic and pregnancy-related factors among women, rural Tanzania, 1995–96

***Variable***	***ANC attendees^a^***	***Maternal deaths Cases 1^b^***	***OR^c ^(95%CI)***	***Maternal deaths Cases 2^d^***	***OR^c ^(95%CI)***
***Total ^e^***	135	45		31	
***Age***					
15–24 years	65	14	1.0	9	1.0
25–34 years	56	18	1.5 (0.7–3.3)	12	1.6 (0.6–4.0)
35–49 years	14	12	**4.0 (1.5–10.6)**	9	**4.1 (1.3–12.4)**
***Ward groups ^f^***					
1	54	4	1.0	3	1.0
2	6	7	**18.0 (2.6–123)**	6	**17.2 (2.1–143)**
3	27	11	1.4 (0.3–6.9)	6	1.8 (0.3–10.8)
4	38	15	2.8 (0.7–11.7)	13	3.3 (0.6–17.1)
5	5	5	**23.1 (2.0–271)**	1	**30.1 (1.1- 823)**
6	5	3	7.6 (0.8–76.7)	2	4.5 (0.3–69.6)
***Woman's ethnic affiliation***					
Iraqw	111	31	1.0	23	1.0
Datoga	22	8	1.3 (0.5–3.3)	5	1.2 (0.4–3.7)
Other	2	6	**13.6 (2.5–75.0)**	3	**7.3 (1.1–50.0)**
***Husband's ethnic affiliation***					
Iraqw	95	29	1.0	21	1.0
Datoga	25	9	1.3 (0.5–3.1)	8	1.6 (0.6–4.2)
Other	4	2	2.3 (0.4–13.7)	1	1.5 (0.2–14.4)
***Woman's religious affiliation***					
Christian	104	27	1.0	19	1.0
Traditional/other	30	18	**2.1 (1.0–4.5)**	12	2.0 (0.8–4.9)
***Husband's religious affiliation***					
Christian	84	18	1.0	11	1.0
Traditional/other	38	22	**2.6 (1.2–5.7)**	19	**3.6 (1.5–8.7)**
***Woman's education***					
1–7 years school	96	24	1.0	18	1.0
No school	38	15	1.2 (0.5–2.6)	12	1.3 (0.5–3.1)
***Husband's education***					
1–7 years school	92	20	1.0	16	1.0
No school	32	17	**2.2 (1.0–5.0)**	12	2.0 (0.8–5.1)
***Parity***					
Pregnancy 1	36	10	1.5 (0.6–3.9)	8	1.9 (0.6–5.7)
Pregnancy 2–4	56	16	1.0	10	1.0
Pregnancy 5–15	43	17	0.5 (0.2–1.5)	13	0.7 (0.2–2.3)
***Previous abortions***					
No abortions	115	41	1.0	30	1.0
1–3 abortions	20	2	0.2 (0.04–1.0)	1	0.2 (0.02–1.2)
***Previous stillbirths***					
No stillbirths	122	38	1.0	26	1.0
1–2 stillbirths	13	5	1.1 (0.4–3.3)	5	1.7 (0.5–5.3)
***Previous perinatal deaths***					
No perinatal deaths	110	34	1.0	24	1.0
1–3 perinatal deaths	25	9	0.9 (0.4–2.3)	7	1.2 (0.4–3.1)

^a ^Antenatal clinic attendees (control group)

^b ^Cases 1 – all maternal deaths found in the study

^c ^Adjusted for age and ward (place of residence)

^d ^Cases 2- all maternal deaths of women who attended any antenatal clinic during last pregnancy

^e ^There were various numbers of missing cases and controls in the different groups

^f ^Wards grouped according to location: 1 = Haydom (reference), 2 = Dongobesh+Tumati+Bashay; 3 = Basotu+Basodesh; 4 = Maghang+Maretadu; 5 = Laghanga+Getanwas+Hirbadaw; 6 = Yaeda Chini.

Parity in this context includes all known abortions and pregnancies, including the current pregnancy when interviewed, as referred by relatives or women being interviewed. Early abortions would in general not be recognised by family, thus abortions would often be late ones, rendering the variable as parity rather than gravidity.

All variables were entered as categorical. In addition, in the analysis, all variables and among them potential confounders (woman's ethnic origin, woman's and husband's religious affiliation, husband's education, and parity) were adjusted for, together and with separate adjustment for each.

Thirty-one of the women who died, had attended antenatal clinics, while fourteen had not, whereas the control group consisted of only antenatal clinic attendees. Thus, in order to assess a possible selection bias, we performed parallel analyses on a sub-set of the cases, comprising the 31 women who had attended antenatal clinics (table 1).

## Results

### Demographic characteristics

The mean age among the cases was 29.1 years (range 18 to 49), and among the controls 26.1 years (range 16 to 41). In multivariate logistic regression analysis with adjustment for ward, there was a significantly increased risk of maternal death in the age group 35–49 years (OR 4.0; 95%CI 1.5–10.6) compared to the group of 25–34 years (table 1). This increased risk persisted after adjustment for the variables mentioned above (woman's ethnic origin, woman's and husband's religious affiliation, husband's education, and parity), and when we restricted analyses to the sub-group who had attended antenatal clinics.

In order to assess whether the age-groups of the controls were representative, we compared the age distribution in the control group with that among all the antenatal attendees at the same four clinics, and also with women of reproductive age in the general population (table 2). The distribution of age was similar for all age groups, except for a slight over-representation in the 15–24 year group and a slight under-representation in the two other age groups.

**Table 2:** Age distribution among pregnant women and women of reproductive age in study area, 1995–96

***Group represented***	***15–24 years***	***25–34 years***	***35–49 years ***^a^
	***% (n)***	***% (n)***	***% (n)***
Cases (maternal deaths)	31.1% (14)	40.0 % (18)	26.7 % (12)
Control group	48.6% (65)	41.5% (56)	10.4% (14)
Four antenatal clinics in study ^b^	43.4% (858)	45.0% (890)	11.5% (228)
Estimated number of women giving live births ^c^	37.5 % (1824)	47.5 % (2310)	15.0 % (732)

^a^The 1988 census [24] categorised women from 45–54 years, and numbers for the age-group 45–49 were not given. Therefore, in the census group the age-span denoted 35–49 years includes only women aged 35–44. In the group of randomly chosen 135 ANC controls, no woman (1 missing) was in the age-group from 45–49 years.

^b ^Four antenatal clinics were chosen for a study of anaemia. The control group in the present study was randomly selected from this group. One woman was 14 years old, but included in the age-group of 15–24 years. 16 women (0.8%, 136 missing) were in the age-group of 45–49 years.

^c ^Based on the 1988 Census [24]and a household survey [25] the number of estimated live births were found by multiplying the women of reproductive age in each group by the age-specific fertility rate/1000.

By categorising wards according to geographic location, we found that residents of some wards had a significantly increased risk of maternal death (OR 18.0; 95% CI 2.6–123 and OR 23.1; 95%CI 2.0–271). The wide confidence intervals indicate that the risk is merely an approximation, and is due to small numbers. This persisted when adjusting to all variables, together and separately, which were included in the analyses. Hence we adjusted for ward in all the analyses.

Among the cases, 69% were Iraqw, 18% Datoga, and 13% came from various other ethnic groups, not indigenous to the study area. For the controls there were 82% Iraqw, 16% Datoga and 1.5% from other groups. There was a significantly increased risk of maternal death among the women not belonging to the Iraqw or Datoga ethnic groups (OR 13.6; 95%CI 2.5–75.0) (table 1), which prevailed after adjustment for selected variables and when the analysis was restricted to the sub-group of cases who had attended antenatal clinics.

There was a significantly increased risk of maternal death among the women who were affiliated with the traditional indigenous beliefs (OR 2.1; 95%CI 1.0–4.5). Traditional indigenous beliefs were defined as those being part of the ethnic culture from before the exposure to any of the foreign major world religions, such as Islam, Christianity, Hinduism, Judaism and others. There were no Islamic men and only three Islamic women, two among the cases and one among the controls. When restricting analyses to those cases attending antenatal clinics and adjusting for the other selected variables, a similar point estimate was observed, but not statistically significant.

Husbands had a traditional religious affiliation in 55% of the maternal death cases against 31% of the controls. The difference was significant (OR 2.6; 95%CI 1.2–5.7) (table 1). When adjusting for selected potential confounders, both together and separately, the significantly increased risk persisted.

Thirty-nine per cent of the cases and 28% of the controls had no formal education. The woman's educational level was not significantly associated with increased risk of maternal death (table 1), whereas a significantly increased risk was observed with husband's education (OR 2.2; 95%CI 1.0–5.0). When adjusting all other variables showing significant associations in table 1, the risk was still increased, but no longer statistically significant.

Virtually all cases and controls were married. Marital status and the husbands' ethnic affiliation were not associated with an increased risk of maternal death.

### Pregnancy-related characteristics

The median number of pregnancies was four (range 1–13) for cases, and three (range 1 to 12) for controls. The point estimate for primiparas showed an increased risk of maternal death, but not statistically significant. There was no increased risk of death for multiparous women. There was no significantly increased risk of maternal death in analyses of previous abortions, stillbirths or perinatal deaths (table 1).

## Discussion

We found an increased risk of maternal death among women over 35 years of age and among couples with a traditional religious affiliation. Further, the risk was increased among women coming from ethnic groups other than the ones indigenous to the area, and among women whose husbands lacked formal education. The strength of the study is the comprehensive health-facility and population-based information on maternal deaths. Since maternal deaths are relatively rare events, the sample size was small, and weaker risk factors may not have been detected.

### Methodological considerations

This study was not originally designed as a case-control study. We decided to use the available data in order to assess risk factors for maternal deaths, since the two studies from where the data originated were ongoing in the same population at the same time. Further, both projects used the same set of questions related to socio-demographic characteristics and had the same principal investigators.

Age, antenatal clinic attendance and place of residence could represent potential sources of selection bias and confounding. In relation to place of residence (ward), the cases were traced through the whole study area, while the controls were self-reporting at the four selected antenatal clinics in the study area, with those living closest probably most likely to attend. The controls represented 11 of 12 possible wards, and we minimised this potential selection bias by adjusting for ward. Further, in relation to antenatal clinic attendance, we attempted to reduce the selection bias by analysing the sub-set of cases that had attended antenatal clinics. In general, the associations found in the total series were upheld in these sub-set analyses.

In relation to age, table 2 shows that the women of the younger age group were slightly over-represented, while the two other age groups were slightly under-represented, thereby possibly creating selection bias. By adjusting for age, we attempted to minimise the effect of this bias. Many of the women did not know their date of birth. Thus, the age recorded was approximate for many of them.

Lastly, the result may be influenced by recall bias. Among the cases in our study, the respondents were relatives of the deceased women, whereas the controls responded personally. This may have led to an information bias by misclassification among cases. For some variables, the events were more noticeable, thus being hard data that were easy to recall. Some events may have gone unnoticed by others than the women themselves. As an example, the controls would most likely recall whether they had aborted or not, whereas the families of the deceased women may not have remembered or known about abortions. Such under-reporting of abortions among cases might have led to what might appear as a protective effect.

### Age and parity

Women in the age group of 35–49 years had a significantly increased risk of maternal death. An under-representation of older women in the control group may have contributed to a slight over-estimation of the risk in this group, but the effect of age remained in the analysis, when we included only those who had attended antenatal clinics in the case group.

The older women appeared to have an increased risk, independent of parity. This may not necessarily be explained by obstetric factors alone, but may also be due to other factors. There could be a decrease of the general state of health among older women, or less active health-seeking behaviour, rendering them more at risk. This seems reasonable when we look at the causes of death among the cases. Of seven women struck by maternal death in the older age group, six died of malaria, while one died of other indirect obstetric causes. All had five or more pregnancies. In addition, younger women might have had husbands of higher educational level or with less reluctance to using modern health facilities.

Loudon [15] investigated the relationship between age and parity, and showed that for all ages, there was a higher risk of maternal death in first births than in second or third. From the fourth birth onwards, increasing parity led to higher maternal mortality regardless of maternal age. Likewise, for all parities, maternal mortality increased with age, but the effect was strongest on first births and fourth and higher order births. With regard to age in settings similar to the current study, some authors have found significant associations between increasing age and risk of maternal deaths in rural districts [16,17], while Urassa *et al*. [8] in a study from the city of Dar es Salaam, and a sentinel surveillance study from both rural and urban districts in Tanzania, did not [14]. Further, several studies found increased risk of maternal death with increasing parity [8,16]. Others observed that primiparas and women with parities of higher order were at increased risk of maternal death [17,18,28]. Our data are compatible with increased risk for primiparas, although it is not statistically significant.

### Ethnic and religious affiliation

We observed an increased risk in women from ethnic groups other than those indigenous to the area. In general, there were few women from other ethnic groups residing in the study area at the time of the study, and the small sample sized resulted in wide confidence intervals. Only women who were residents of the study area were included in the study. Many factors may affect and explain the results related to ethnicity, such as discriminatory practices and injustices in society, cultural rituals connected to pregnancy and birth, lack of social network, and poverty. The Iraqw and Datoga have distinctly differing practices on reproductive issues, and both differ significantly from the Bantus in the study area [21,29]. Most of the study area population were small-scale subsistence farmers or pastoralists. There was no industrial activity in the area.

There are several potential explanations of the apparently increased risk of women and husbands who adhered to religions other than Christianity. Perhaps they all relate to culture, which again may be influenced by both religion, economy, ethnicity, and geographical location. In this society, people of all religions lived closely integrated, with intermarriage being common. If the husband had a traditional belief and the woman was a Christian, the husband would still probably be the decision-maker, making him the most influential as to health decisions. The hospital, health centre, and dispensaries in the study area were all part of the National Health plan and thus catered to all groups, regardless of belief. From our knowledge of the area, there was an extensive use of traditional medicine by all religious groups. Distance to a hospital may in itself be a risk factor, thus being a possible confounder to religion. When adjusting for ward, we found that the risk persisted. In the wards with the highest risks, there were dispensaries and a health centre. These did not offer comprehensive obstetric care.

Women with a Catholic affiliation were found to have a significantly increased risk of maternal death in an urban area in Zimbabwe [16], while a similar study from Dar es Salaam did not identify religion as a risk factor [8]. Thus, we can give no clear explanation for the findings on religion, and it may all perhaps be related to aspects of culture.

### Education

The husbands' lack of formal education appeared to be a risk factor. A study in India found a significant association between a low level of husbands' education and the risk of maternal death [17]. Similarly, in Nepal, if the husband had a level of education greater than fifth grade, it significantly reduced the high-risk practices in all stages of childbirth, independent of other socio-economic, biological and village variables [30]. In a study from Kenya, in households headed by males, although the women were significantly less likely to deliver at health facilities, the educational level of the household head was not significantly associated with place of delivery [20].

It is important to consider the role and power of the decision-makers in societies where the risk of maternal deaths is high. A study from Dar es Salaam observed that when complications arose, the mother of the woman decided where to seek help in 31% of the cases, while the husband decided in 29% of the cases. Only in 5% of the cases did the deceased women make their own decision [31]. The importance of the husbands' level of knowledge may perhaps be linked to the fact that in many societies in developing countries, husbands are the decision-makers. We know that some women in the present study dared not go to the hospital or other health facility without their husband's explicit permission. When husbands were not at home at the time of delivery, this could have serious adverse effects.

In our study, the women's education did not seem to be associated with an increased risk of maternal death. Previous studies have shown conflicting results, where some found similar results to ours [16,17], while others found that maternal education does exert an effect [8,18,19,32]. Our study may have had too few participants to detect a real association.

A great effort has been made to educate women about health matters through the antenatal clinics in many countries. In Tanzania, antenatal attendance was 92 % at the time of the study [33], and lectures on health related matters are among the activities at the clinics. It is important that the women have separate settings where they can feel free to ask questions. However, there may be a case for educating males as well as women, in order to influence decisions related to health. Although we measured the risk of maternal death in relation to formal school education, we advise health workers to hold educational sessions on health-related issues, delivery- and complication-preparedness, since every pregnancy is potentially at risk of complications. Teaching by males for males is important at places where men gather, such as village meetings, bars, and local clubs, in addition to an increased focus on making primary school education available to even more children, both females and males.

### Obstetric history

We did not observe significantly increased odds ratios for maternal death among women who had previously experienced stillbirths and perinatal deaths. Loss of children, either as abortions, stillbirths, or neonatal loss, has been shown in other studies to represent an increased risk of maternal death [8,16]. The power of our study may have been too low to detect effects of low magnitude. On the other hand, the recall bias mentioned in relation to obstetric factors in general and abortions in particular, would probably not have had a major effect on these two variables.

## Conclusion

Lack of formal education of husbands was associated with an increased risk of maternal death. This may reflect the role of the decision-makers in a household, leading to different health seeking behaviour among those women whose husbands had a higher education. Further, maternal death was associated with increased maternal age, and to religious and ethnic affiliation. We want to emphasise the importance of improved access for delivering women to comprehensive obstetric care, and investing in increased health education of men, especially in relation to pregnancy and delivery, in addition to the education of women. Further, investing in research on the role of males in relation to health-related issues may be valuable for understanding risk factors associated with maternal deaths in developing countries.

## Competing interests

The author(s) declare that they have no competing interests.

## Authors' contributions

BEO and SGH designed the study, were the principal investigators and did the data collection. Further they supervised the data entry and analysed all the data. BEO drafted the manuscript. In addition to above mentioned activities, SGH commented on all drafts of the manuscript. RTL supervised the statistical analysis and commented on all drafts. PB supervised the data collection during the field work and commented on all analyses and drafts of the manuscript. PG was a co-investigator during the field work and data collection and had responsibility for all the data entry, in addition to commenting on the drafts of the manuscript. GK was the main supervisor of the project, supervising both the design of the study, the field work, the analyses and the writing process. All authors read and approved the final manuscript.

## Pre-publication history

The pre-publication history for this paper can be accessed here:


